# Graft-versus-host-disease prophylaxis with ATG or PTCY in patients with lymphoproliferative disorders undergoing reduced intensity conditioning regimen HCT from one antigen mismatched unrelated donor

**DOI:** 10.1038/s41409-024-02225-2

**Published:** 2024-02-08

**Authors:** Annalisa Paviglianiti, Maud Ngoya, Marta Peña, Ariane Boumendil, Zafer Gülbas, Fabio Ciceri, Francesca Bonifazi, Domenico Russo, Nathalie Fegueux, Friedrich Stolzel, Claude Eric Bulabois, Gerard Socié, Edouard Forcade, Carlo Solano, Hervé Finel, Stephen Robinson, Bertram Glass, Silvia Montoto

**Affiliations:** 1 Duran i Reynals Hospital, Catalan Institute of Oncology, Barcelona, Spain Institut d’Investigació Biomèdica de Bellvitge (IDIBELL), Barcelona, Spain; 2 Department of Medicine, Unit of Endocrinology and Diabetes, Università Campus Bio-Medico di Roma, Rome, Italy; 3 Lymphoma Working Party, EBMT, Paris, France; 4 Bone Marrow Transplantation Department, Anadolu Medical Center Hospital, Kocaeli, Turkey; 5 Hematology and Bone Marrow Transplant Unit, IRCCS San Raffaele Scientific Institute, Milan, Italy; 6 IRCCS Azienda Ospedaliero-Universitaria di Bologna, Bologna, Italy; 7 Unit of Blood Diseases and Bone Marrow Transplantation, University of Brescia, ASST Spedali Civili of Brescia, Brescia, Italy; 8 Department of Hematology and Oncology, CHU Lapeyronie, Montpellier, France; 9 University Hospital, Dresden, Medizinische Klinik und Poliklinik I, Dresden, Germany; 10 CHU Grenoble Alpes - Université Grenoble Alpes, Service d’Hématologie, Grenoble, France; 11 Hopital Saint Louis, Department of Hematology – BMT, Paris, France; 12 CHU Bordeaux, Hôpital Haut-leveque, Pessac, France; 13 Hospital Clínico de Valencia, Servicio de Hematología, University of Valencia, Valencia, Spain; 14 Department of Haemato-oncology St.Bartholomew’s Hospital, Barts Health NHS Trust, London, UK

**Keywords:** Translational research, Lymphoma

## Abstract

Post-transplant cyclophosphamide (PTCY) has been introduced as graft-versus-host disease (GvHD) prophylaxis in mismatched and matched unrelated hematopoietic cell transplant (HCT). However, data comparing outcomes of PTCY or ATG in patients undergoing a 1 antigen mismatched HCT for lymphoproliferative disease are limited. We compared PTCY versus ATG in adult patients with lymphoproliferative disease undergoing a first 9/10 MMUD HCT with a reduced intensity conditioning regimen from 2010 to 2021. Patients receiving PTCY were matched to patients receiving ATG according to: age, disease status at transplant, female to male matching, stem cell source and CMV serology. Grade II-IV acute GvHD at 100 day was 26% and 41% for the ATG and PTCY group, respectively (*p* = 0.08). Grade III–IV acute GvHD was not significantly different between the two groups. No differences were observed in relapse incidence, non-relapse mortality, progression-free survival, overall survival and GvHD-relapse-free survival at 1 year. The cumulative incidence of 1-year extensive chronic GvHD was 18% in the ATG and 5% in the PTCY group, respectively (*p* = 0.06). In patients with lymphoproliferative diseases undergoing 9/10 MMUD HCT, PTCY might be a safe option providing similar results to ATG prophylaxis. Due to the limited number of patients, prospective randomized trials are needed.

## Introduction

Post-transplant cyclophosphamide (PTCY) has been introduced as graft-versus-host disease (GvHD) prophylaxis for haploidentical hematopoietic cell transplant (HCT) in combination with tacrolimus and micofenolate mofetil in the last decades with significant improvements in outcomes [1]. Giving the encouraging results, the use of PTCY has been extended to matched and, subsequently, to mismatched unrelated HCT with promising results [2, 3]. Several registry studies have reported outcomes of mismatched unrelated donor HCT with PTCY and the addition of immunosuppressive drugs to prevent GvHD in hematological malignancies [4–6]. A recent phase 3 randomized clinical trial comparing PTCY in combination with a short course of cyclosporine A versus the combination of cyclosporine A and mycophenolic acid in matched related and unrelated donor HCT demonstrated a significantly reduction of severe acute and chronic GvHD in the PTCY arm [7]. However, in vivo T-cell depletion with antithymocyte globulin (ATG) in association with a calcineurin inhibitor and mycophenolate mofetil or methotrexate has remained the main strategy in one antigen HLA-mismatched unrelated donor since few years ago (9/10 MMUD) [8–10]. The risk of GvHD is associated with the degree of human leukocyte antigen (HLA) match [8, 11]. Previous published studies have reported a higher incidence of GvHD and non-relapse mortality (NRM) in the setting of MMUD HCT [12]. Recent improvements in supportive care, HLA typing and GvHD prophylaxis, including PTCY, have resulted in better survival outcomes and acceptable toxicity profile after MMUD [13]. In the last years the traditional use of ATG in combination with calcineurin inhibitor and mycophenolate mofetil for 9/10 MMUD HCT has been overtaken by the progressive use of PTCY in this platform in different European institutions. Nevertheless, data comparing the two GvHD prophylaxis in the setting of patients who underwent a MMUD 9/10 HCT are scarce.

A large retrospective study has compared outcomes in this setting for acute myeloid leukemia recipients [14]. All other published studies in 9/10 MMUD HCT included a heterogeneous population with a limited number of patients [15–17].

The aim of this retrospective study is to analyze the outcomes of a homogeneous population of patients with lymphoproliferative disease undergoing 9/10 MMUD HCT with a reduced intensity conditioning regimen and receiving PTCY or ATG as GvHD prophylaxis.

## Materials and methods

This is a retrospective study from the European Society for Blood and Marrow Transplantation Lymphoma Working Party (EBMT-LWP). Data are entered, managed, and maintained in a central database with internet access. Patients provide written informed consent authorizing the use of their personal information for research purposes.

Adult patients (≥18 years old) in all disease status who received a first HCT from a 9/10 MMUD from 2010 to 2021 and for whom high-resolution HLA-allele typing at loci A, B, C, DRB1 and DQ was available in the EBMT data registry were included in the study. A 9/10 MMUD was defined as a difference between the donor and the recipient in a single HLA at HLA-A, B, C, DRB1 or DQ. Performance status (PS) was graded according to the Karnofsky Performance scale. All patients received peripheral blood stem cell as graft source and a reduced intensity conditioning regimen according to EBMT definition [18].

In vivo T-cell depletion (TCD) other than ATG was an exclusion criteria. Patients receiving PTCY were matched to patients receiving ATG according to the following variables: age, disease status at transplant, female to male donor status match, stem cell source and CMV serology.

Patients provided informed consent authorizing the use of their personal information for research purposes. Each patient provided consent for transplant according to the declaration of Helsinki. The study was approved by the Institutional Review Board of the LWP of the EBMT.

The primary endpoint of the study was the cumulative incidence (CI) of GvHD. Acute GVHD (aGVHD) was graded according to the modified Glucksberg criteria [19] and chronic GVHD (cGVHD) according to the revised Seattle criteria [20]. Secondary endpoints included cumulative incidence of engraftment, progression-free survival (PFS), overall survival (OS), graft-versus-host disease/relapse-free survival (GRFS), relapse incidence (RI) and non-relapse mortality (NRM).

Engraftment was defined as achieving an absolute neutrophil count greater than or equal to 0.5 × 10^9^/L for three consecutive days. PFS was defined as the probability of being alive without evidence of relapse or progression. OS was defined as the time from HCT to death, regardless of the cause. GRFS was defined as the time from HCT to grade III-IV aGVHD, severe cGVHD, disease relapse or death from any cause, whichever comes first. Relapse was defined as the time from HCT to reappearance of the underlying disease. Death without evidence of relapse or progression defined NRM.

Categorical variables were compared using the Chi-square or Fischer exact test, while continuous variables were compared with the Mann–Whitney. Probabilities of OS, PFS and GRFS were calculated using the Kaplan-Meier method. Cumulative incidence functions were used to estimate RI and NRM in a competing risk setting. To study GVHD, death and relapse were considered as competing events. Univariate analyses were performed using the log rank test for OS, LFS and GRFS, while the Gray’s test was used for cumulative incidence function. All tests were two-sided and *P* values < 0.05 were considered as statistically significant.

The ATG patients were matched with the PTCY patients at a ratio of 1:2. The matching was done on the following variables: Age, disease status, female to male donor status, cells source and CMV match status. Analyses were performed using the R statistical software version 4.0.2.

## Results

A total of 411 patients were initially identified (*n* = 341 for the ATG group and *n* = 70 for the PTCY group). According to the above mentioned variables, 121 patients receiving ATG were identified and matched with 64 patients receiving PTCY. The baseline characteristics of the pair-matched groups are summarized in Table 1.

**Table 1:** Patient demographics and clinical characteristics at baseline of the pair-matched groups.

	Total *n* = 185	ATG group *n* = 121	PTCY group *n* = 64	*P*-value
Recipient age, years				
Median (range)	52 (19–69)	53 (19–68)	52 (22–69)	0.58
Interquartile range	[40–60]	[41–60]	[38–60]	
Recipient sex, *n* (%)				
Female	67 (36%)	47 (39%)	20 (31%)	0.31
Male	118 (64%)	74 (61%)	44 (69%)	
Diagnosis, *n* (%)				
DLBCL	30 (16%)	21 (17%)	9 (14%)	0.59
FL	23 (12%)	18 (15%)	5 (8%)	
HL	50 (27%)	24 (20%)	26 (41%)	
MCL	20 (11%)	12 (10%)	8 (13%)	
TCL	17 (9%)	13 (10.7)	4 (6%)	
Others	45 (24%)	33 (27%)	12 (19%)	
Disease status at transplantation, *n* (%)				
CR	121 (67%)	79 (67%)	42 (67.7)	1
PR	50 (28%)	33 (28%)	17 (27.4)	
PD/relapse	9 (5%)	6 (5%)	3 (4.8)	
Missing	5	3	2	
Karnofsky, *n* (%)				
Good (≥80%)	171 (96%)	112 (97%)	59 (95%)	0.7
Poor (<80%)	7 (4%)	4 (3%)	3 (5%)	
Missing	7	5	2	
Donor sex, *n* (%)				
Female,	51 (27.7)	33 (27.3)	18 (28.6)	0.85
Male	133 (72.3)	88 (72.7)	45 (71.4)	
Missing	1	0	1	
Female to male donor, *n* (%)	27 (15%)	17 (14%)	10 (16%)	0.77
CMV status donor/recipient, *n* (%)				
Neg/Neg	32 (18%)	21 (18%)	11 (18%)	1
Neg/Pos	37 (20%)	24 (20%)	13 (21%)	
Pos/Neg	6 (3%)	4 (3%)	2 (3%)	
Pos/Pos	108 (59%)	71 (59%)	37 (59%)	
Missing	2	1	1	
Recipient ABO group, *n* (%)				
A	41 (37%)	24 (32%)	17 (47%)	0.47
AB	8 (7%)	6 (8%)	2 (6%)	
B	15 (13%)	10 (14%)	5 (14%)	
O	46 (40%)	34 (46%)	12 (33%)	
Missing	75	47	28	
Recipient Rhesus factor, *n* (%)				
Absent	18 (16%)	9 (12%)	9 (25%)	0.09
Present	92 (84%)	65 (88%)	27 (75%)	
Missing	75	47	28	
DQ HLA mismatch, *n* (%)	38 (21%)	29 (25%)	9 (14%)	0.09
Stem cell source, *n* (%)				
PBSC	185 (100)	121 (100)	64 (100)	Not done
GVHD prophylaxis, *n* (%)				
CsA based	4 (2%)	4 (3%)	0 (0%)	Not done
CsA + MMF based	66 (36%)	34 (28%)	32 (50%)	
CsA + MMF + siro/tacro based	1 (1%)	1 (1%)	0 (0%)	
CsA + MMF + MTX + siro/tacro based	2 (1%)	2 (2%)	0 (0%)	
CsA + MTX based	61 (33%)	61 (50%)	0 (0%)	
PTCY alone	1 (1%)	0 (0%)	1 (2%)	
Without CsA	50 (27%)	19 (16%)	31 (49%)	
Year of HSCT, *n* (%)				
2010–2015	81 (44%)	71 (59%)	10 (16%)	Not done
2016–2021	104 (56%)	50 (41%)	54 (84%)	

*ATG* antithymocyte globuline; *PTCY* post-transplant cyclophosphamide, *DLBCL* diffuse large B-cell lymphoma, *FL* follicular lymphoma, *HL* Hodgkin lymphoma, *MCL* mantle cell lymphoma; *TCL* T-cell lymphoma, *CR* complete response, *PR* partial response, *PD* progressive disease, *HCT-CI* hematopoietic cell transplant comorbidity index, *CMV* cytomegalovirus, *Neg* negative, *Pos* positive, *PBSC* peripheral blood stem cells, *GVHD* graft-versus host disease; *CsA* cyclosporine A, *MMF* mycophenolate mofetil, *Siro* sirolimus; Tacro: tacrolimus, *MTX* methotrexate, *HSCT* hematopoietic stem cell transplant.

The majority of patients underwent a previous autologous HCT (62% in the ATG and 65% in the PTCY group, respectively) and presented a mismatch at either HLA-A, B, C, DRB1 (*n* = 147; 89%)

The results of the main survival outcomes after matched-pair analysis are summarized in Table 2.

**Table 2:** Outcomes after pair-matched analysis in ATG and PTCY group.

	ATG group (95% CI)	PTCY group (95% CI)	*P*-value
1-year OS (%)	62 (52–71)	55 (39–69)	0.70
1-year PFS (%)	57 (47–66)	50 (34–64)	0.74
1-year NRM (%)	22 (15–30)	34 (20–49)	0.12
1-year RI (%)	21 (14–30)	16 (7–29)	0.19
Neutrophile engraftment at day +30 (%)	97 (91–99)	100	0.09
Grade II-IV aGVHD at day +100 (%)	26 (18–35)	41 (28–54)	0.08
Grade III-IV aGVHD at day +100 (%)	11 (6–18)	15.7 (7.3–27)	0.68
cGVHD at 1 year (%)	29 (20–38)	20 (9–34)	0.51
Extensive cGVHD at 1 year (%)	19 (11–27)	5 (1–15)	0.06
1-year GFRS (%)	41 (31–50)	47 (31–61)	0.68

*ATG* antithymocyte globuline, *PTCY* post-transplant cyclophosphamide, *95% CI* 95% confidence interval, *OS* overall survival, *PFS* Progression-free survival, *NRM* non-relapse mortality, *RI* Relapse incidence, *aGVHD* acute graft-versus-host disease, *cGVHD* chronic graft-versus-host disease, *GFRS* graft-versus-host disease-free, relapse-free survival.

### Engraftment, acute and chronic GvHD

The cumulative incidence of neutrophil engraftment was 98% (CI: 94–99.5). It was 97% (CI: 91–99) and 100% in ATG and PTCY group, respectively (*p* = 0.09).

**Fig. 1:**
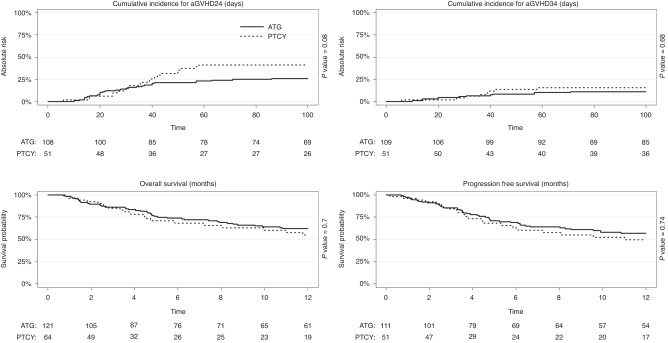
Cumulative incidence of acute GvHD, OS and PFS for ATG and PTCY group. Cumulative incidence of grade II–IV acute GvHD (1), cumulative incidence of grade III-IV acute GvHD (2), OS (3) and PFS (4) for ATG (continuous line) and PTCY (dashed line) group.

Overall, the 100-day cumulative incidence of grade II–IV acute GvHD was 31% (CI: 24–38) and there were not statistically significant differences according to the GvHD prophylaxis group. The cumulative incidence of grade III–IV acute GvHD was 13% (CI: 8–18) at 100 day. It was not statistically different between the group of patients who received ATG and those who received PTCY (11% [CI: 6–18] versus 16% [CI: 7–27], *p* = 0.68) (Fig. 1). The cumulative incidence of 1-year chronic GvHD and extensive chronic GvHD was 26% (CI: 19–34) and 15% (9–21), respectively.

The cumulative incidence of 1-year chronic GvHD and extensive chronic GvHD was also comparable between the two groups (Table 2). According to GvHD prophylaxis, the cumulative incidence of chronic GvHD was 29% (CI: 20–38) and 20% (CI: 9–34) in patients who received ATG and PTCY, respectively (*p* = 0.51). The cumulative incidence of extensive chronic GvHD was also comparable between the two groups, being 19% (CI: 11–27) in the ATG group and 5% (CI: 1–15) in the PTCY group, respectively (*p* = 0.68).

### RI and NRM, OS, PFS and GRFS

**Fig. 2:**
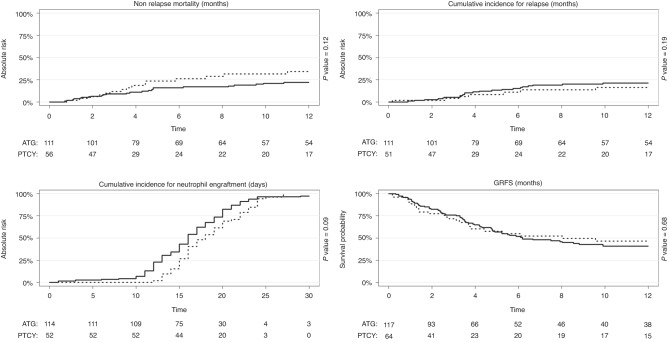
Cumulative incidence of NRM, RI, neutrophil engraftment and GRFS by ATG and PTCY. Non relapse mortality (1), relapse incidence (2), neutrophil engraftment (3) and GRFS (4) in ATG ATG (continuous line) and PTCY (dashed line) group.

RI at 1 year was 20% (CI: 14–27). No differences were observed in 1 year RI between the ATG and PTCY group (*p* = 0.19). NRM was 26% (CI: 19–33) at 1 year. NRM was not statistically different between the two groups (*p* = 0.12) (Fig. 2).

PFS and OS at 1 year were 55% (CI: 46–63) and 60% (CI: 52–67), respectively. PFS, GRFS and OS were not different across the two groups (Table 2) .The median follow-up was 6 (CI: 4–7) years for the ATG group and 1 (CI 0.5–2) years in the PTCY group (*p* < 0.001).

Fifty-three patients died in the ATG group, 21 died in the PTCY group. Death was mainly due to transplant related complications (*n* = 31 in ATG and *n* = 15 in PTCY patients, respectively) followed by relapse of disease (*n* = 15 in ATG and *n* = 4 in PTCY group).

## Discussion

We observed comparable outcomes in patients with lymphoproliferative disease undergoing a reduced intensity conditioning regimen based 9/10 MMUD HCT when comparing ATG and PTCY based GvHD prophylaxis. The rates of any grade acute and chronic GvHD were comparable with the use of PTCY versus ATG.

In the setting of a one-antigen mismatch HCT, as we know for haploidentical HCT, intensification of GvHD strategies is important in order to overcome the HLA barrier. Nevertheless, there is no agreement regarding the optimal approach for GvHD prophylaxis in 9/10 MMUD HCT. Moreover, other variables should be considered when evaluating outcomes and the risk of developing GvHD, such as stem cell source, CMV serology and gender matching. Therefore, to minimize the influence of confounding variables and ensure comparability between the two groups, we conducted a matched-paired analysis in the present study. This approach considered factors that primarily affect GvHD.

Traditionally, ATG has been the preferred choice of treatment in this setting since it can partially overcome the HLA barrier. However, its use has been associated to an increased risk of infections, delayed immune reconstitution and relapse. More recently, PTCY has been implemented in other contexts apart from haploidentical HCT, demonstrating being a feasible approach. Several prospective studies have reported the effectiveness of HCT with a MMUD in the setting of PTCY indicating that this approach may significantly expand access to HCT [7, 21].

In our retrospective cohort, we observed an unbalanced distribution of the immunosuppressive agents associated with either ATG or PTCY, with the majority of patients in the PTCY arm receiving MMF and CsA and the majority of those in the ATG group receiving CsA and MTX. To date, the question of the best combination remains unanswered with differences according to center policies still being important, as revealed by our multicenter retrospective study.

The effects of different mismatch types on MMUD HCT outcomes have been well-documented [22]. In the setting of a one antigen MMUD HCT, the increased incidence of acute GvHD following PTCY could be attributed to a higher degree of minor antigen mismatches and less effective in vivo elimination of effector T cells [23].

Two previous studies have reported a lower incidence of acute GvHD in patients receiving PTCY. In contrast to our report, the aforementioned studies were performed on both myeloid and lymphoid malignancies receiving a myeloablative and reduced intensity conditioning regimen [16, 17]. In the first mentioned study only 15% of patients (*n* = 11 in ATG and *n* = 9 in PTCY) were 9/10 MMUD which is a limited number of patients preventing to draw conclusions and comparisons in this case [17]. The second one compared patients receiving lower doses of PTCY of 40 mg/kg (*n* = 22) to patients receiving ATG (*n* = 58) and found significantly lower rates of grade II-IV GvHD and higher OS when using PTCY [16]. Both studies included a limited number of patients with lymphoproliferative disease (*n* = 10 and *n* = 28, respectively).

Another published study by Metha et al. reported lower GvHD rates in the PTCY group when compared to ATG. The study was performed on 113 patients receiving myeloablative or reduced intensity conditioning regimen and of whom 52 were lymphoid malignancies. In contrast to our study, the graft source used was bone marrow in the majority of the cases [15]. Nevertheless, we observed a similar incidence of grade III-IV acute GvHD of 12% and 17%, while they observed a non-significant difference in grade II-IV acute GvHD (37% versus 36%) at 100 day.

Other studies have reported ATG versus PTCY GvHD prophylaxis approach in the myeloid setting with a reduced or myeloablative conditioning regimen. Modi et al. reported the outcome of 76 patients with myeloid malignancies undergoing a 7/8 MMUD HCT with ATG (*n* = 51) or PTCY (*n* = 25) as GvHD prophylaxis [24]. The results show no difference in grade III–IV acute GvHD, but higher grade II-IV acute GvHD and higher 1-year chronic GvHD in the ATG group. However, the characteristic of the study (being a single center study), the product and dose of ATG considered, the type of disease included and the HLA matching definition might have contributed to the differences in the GvHD rates.

A more recent publication in 272 patients with acute myeloid leukemia undergoing myeloablative or reduced intensity conditioning regimen showed lower grade III–IV acute GvHD in the PTCY group, with no difference in any other grade of chronic GvHD nor in acute grade II-IV GvHD [14]. Patients who received PTCY experienced a significantly higher probability of leukemia free survival when compared to patients receiving ATG [14]. These findings are in part in line with ours for chronic GvHD but we observed a difference in acute GvHD rates. The different type of disease included and of conditioning regimen (more frequently used a reduced conditioning in the setting of lymphoid malignancies) might have played a role preventing to draw conclusions.

Compared to the other previously published reports, our study is the only one focusing not only on a homogeneous cohort of patients with lymphoproliferative disease but also on a cohort receiving a reduced intensity conditioning.

Along with a comparable acute GvHD we also observed similar rates of chronic GvHD in both ATG and PTCY groups. This finding may be explained by the mechanism of action of ATG, which acts not only with an extensive in vivo T-cell depletion and with expansion of regulatory T cells, but by also targeting B cells [25, 26]. Given that both donor T and B cells are crucial in the development of chronic GvHD, ATG prolonged inhibitory effect on these cells may help in preventing this complication in the long term [27]. The effects of different mismatch types on MMUD HCT outcomes have been well-documented [22]. In the setting of a one antigen MMUD HCT, the increased incidence of acute GvHD following PTCY could be attributed to a higher degree of minor antigen mismatches and less effective in vivo elimination of effector T cells [23].

We observed similar progression and overall survival between the two groups, and rates of relapse and NRM, which is in line with previous reports [14–17, 24]. On the contrary, a lower NRM for patients receiving a PTCY based GvHD prophylaxis has been reported in the latter one [14], but maybe due to the limited number of patients in our cohort we were not able to observe any difference between the two GvHD prophylaxis groups.

We acknowledge certain limitations in our study. Being a retrospective multicenter study, we were not able to report some parameters such as the dose of ATG, or other parameters which could have helped to determine the benefits of PTCY in patients with HLA class I versus class II mismatches. Furthermore, the follow-up of the PTCY group was shorter than ATG group. Nevertheless, the homogeneity of the group with respect to conditioning intensity, disease type and graft source and the matching paired analysis ensured to avoid some bias.

## Conclusions

In patients with lymphoproliferative diseases undergoing 9/10 MMUD HSCT, PTCY as GVHD prophylaxis might be a safe option providing similar results to ATG prophylaxis. Due to the limited number of patients, prospective randomized trials are needed to allow better understand the effectiveness of adding PTCY into different platforms, including MMUD 9/10 HSCT.

## Data Availability

Data sharing would only be considered for research purposes after specific requests and internal revision and consideration.
